# Improvement of Giant Cell Lesions of the Jaw Treated With High and Low Doses of Denosumab: A Case Series

**DOI:** 10.1002/jbm4.10010

**Published:** 2017-07-25

**Authors:** Tara S Kim, Gianina L Usera, Salvatore L Ruggiero, Stuart A Weinerman

**Affiliations:** ^1^ Division of Endocrinology Diabetes and Metabolism Hofstra Northwell School of Medicine at Hofstra University Manhasset NY USA; ^2^ New York Center for Orthognathic and Maxillofacial Surgery Lake Success NY USA; ^3^ Division of Maxillofacial Surgery Hofstra Northwell School of Medicine Manhasset NY USA; ^4^ Department of Oral and Maxillofacial Surgery Stony Brook School of Dental Medicine Stony Brook NY USA

**Keywords:** TUMOR‐INDUCED BONE DISEASE, CANCER, STROMAL/STEM CELLS, CELLS OF BONE, DENTAL BIOLOGY, ANTIRESORPTIVES, THERAPEUTICS

## Abstract

Giant cell tumors (GCTs) and central giant cell granulomas (CGCGs) are aggressive lesions that appear in the jaw. These lesions occur in the second and third decades of life and often arise in the mandible. Clinical manifestations of these lesions vary from asymptomatic to symptomatic tooth displacement with cortical perforation. GCTs, which are characterized by multinucleated osteoclast‐type giant cells that express receptor activator of nuclear factor‐κB (RANK) ligand, rarely present in the jaw and have overlapping histopathologic features with CGCGs, which are composed of fibroblastic stromal cell lesions. GCTs and CGCGs have overlying histopathologic features that make distinction between the two challenging. There is a real controversy as to whether giant cell tumors and central giant cell granulomas are in fact, one and the same lesion. The majority of GCTs occur in the long bone, with surgery being the typical therapeutic option. Denosumab as a treatment modality is a fairly new concept that has been used effectively in GCTs affecting long bones. There is less experience, however, with its use for jaw lesions. This seven‐case series describes the effective use of both low‐dose and high‐dose denosumab in the treatment of GCTs and CGCGs affecting the jaw and special dosing considerations for younger patients who present with disease. © 2017 The Authors. *JBMR Plus* Published by Wiley Periodicals, Inc. on behalf of the American Society for Bone and Mineral Research.

## Introduction

Giant cell tumors (GCTs) and central giant cell granulomas (CGCGs) are similar appearing lesions that appear in the jaw. CGCG are lesions unique to the jaws and are classified as reactive lesions that may behave as a neoplasm. They occur in the second and third decades of life with a predilection for the mandible. Most patients are asymptomatic, although tooth displacement and expansion with cortical perforation may occur in more aggressive lesions.^1^ GCTs of bone, generally considered a separate entity from CGCGs, are lesions characterized by multinucleated osteoclast‐type giant cells that express receptor activator of nuclear factor‐κB (RANK) and mononuclear stromal cells that express RANK‐ligand (RANKL).^2^ GCTs are rarely present in the jaws but have overlapping histopathologic features with CGCG that make distinction between the two lesions challenging.

CGCGs have varying microscopic patterns that range from vascular to fibrotic to myxoid stroma. The dominant stromal cells are fibroblastic. The giant cells in CGCGs are CD68‐positive and vary in size, shape, and number.^1^ Comparatively, histologic features that favor the diagnosis of GCT over CGCG include sheets of neoplastic ovoid mononuclear cells with high RANKL expression, RANK‐positive mononuclear cells of myeloid lineage, and large RANK‐expressing osteoclast‐like giant cells.^3^ Although there has been a distinct delineation between GCTs and CGCGs based on the aggressive behaviors of GCTs, clinical and histomorphologic data suggest that GCT and CGCG represent a spectrum of a single disease process modified by patient age and site of lesion occurrence.^4^

Traditionally, surgery has been the treatment modality for both pathologic entities. However, high recurrence rates and morbidity associated with resection render it a suboptimal treatment choice. We present a case series of one GCT of the jaw, five CGCG of the jaw, and one case of giant cell lesion consistent with cherubism, all of which were referred to endocrinology to determine whether medical therapy could obviate the need for planned surgical resection. Denosumab, a fully human monoclonal antibody that inhibits RANKL,^5^ was approved by the US Food and Drug Administration (FDA) in June 2013 for the treatment of un‐resectable GCTs of bone in adults and skeletally mature adolescents. Our primary endpoint in this series was to identify whether surgical management for these progressive lytic lesions could be eliminated with the use of denosumab. Our secondary endpoint was to monitor radiographic improvement while on treatment.

## Patients and Methods

### Case 1

A 20‐year‐old woman with Noonan syndrome—a genetic disorder that presents with short stature, distinctive facial features, chest deformity, and congenital heart disease^6^—was diagnosed with giant cell tumor of the bone (GCT) localized to the ramus regions of the jaw in 2002. The tumor was found on exam by an otolaryngologist who was treating the patient for an acute bout of sinusitis. She was referred to an oral surgeon who subsequently biopsied the lesion and confirmed a diagnosis of GCT. Despite six dental extractions, deemed necessary in the context of the expansile and lytic nature of the lesion, and an 18‐month course of subcutaneous calcitonin 100 IU daily, she developed progressive disease.

**Figure 1:**
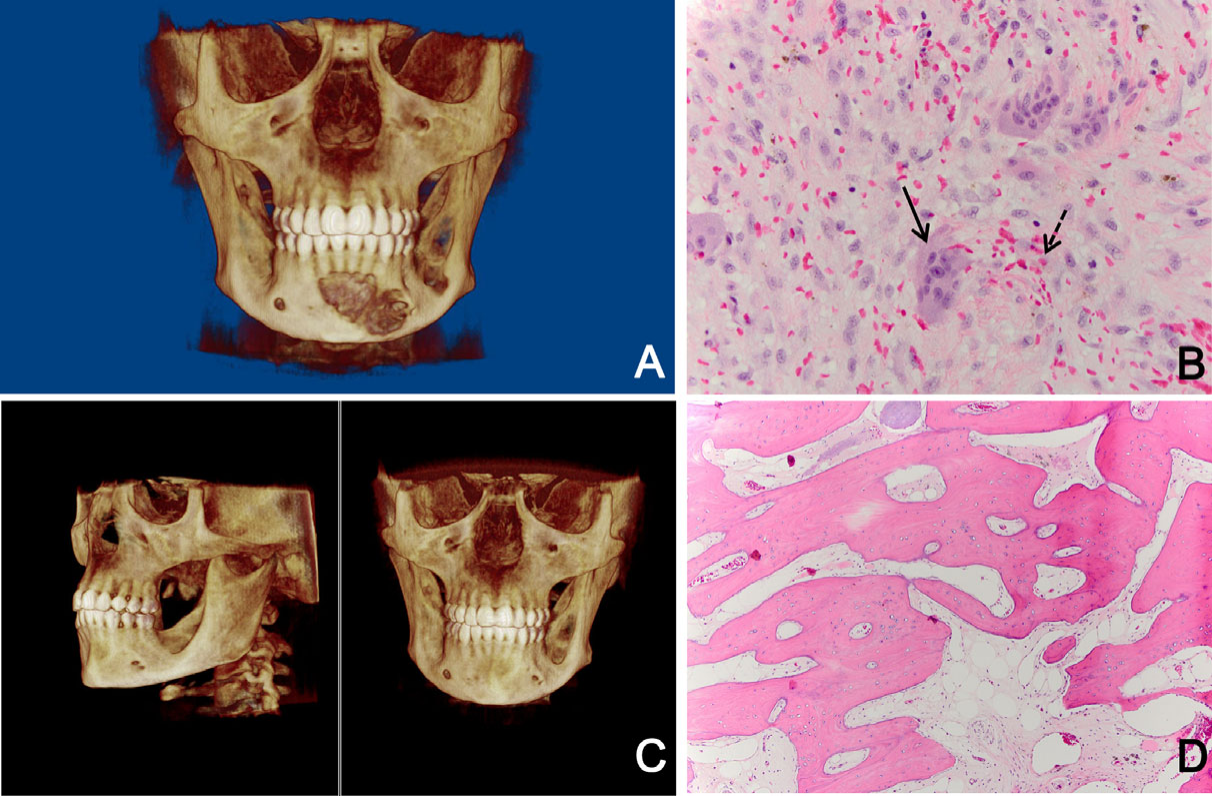
(*A*) Jaw X‐ray showing an expansive mandibular lesion with irregular borders. (*B*) Biopsy showing central lesion (solid arrow) with perivascular hyalinization (dashed arrow). (*C*) Panoramic X‐ray 1 year on treatment shows resolution of the lesion. (*D*) Biopsy confirmed viable lamellar and trabecular bone with fibrous connective tissue. No giant cell lesion observed.

The patient had no personal or family history of metabolic bone disease and did not receive any growth hormone for her short stature. She reached an adult height of 5 feet 0 inches. In March 2002, imaging showed a new mandibular lesion in the anterior mandible with irregular borders measuring approximately 28.4 mm (width) × 21.8 mm (height). Biopsy showed central giant cell lesions with associated perivascular hyalinization (Fig. 1 *A*, *B*). Initial workup showed a 25‐OH vitamin D level of 9.8 ng/mL and ergocalciferol 50,000 units weekly was started. N‐terminal telopeptide of type 1 collagens (NTx) was 48 nM BCE/mM creatinine with normal range being 4 to 64 nM BCE/mM creatinine in premenopausal females. PTH, bone‐specific alkaline phosphatase, and phosphorus were within expected reference ranges.

#### Treatment

Based on the patient's age and the lack of published data specifically addressing GCT affecting the jaw, we opted to begin at conservative doses while monitoring for any potential adverse reactions. The decision was made to start treatment with a lower dose of subcutaneous denosumab (60 mg monthly) as opposed to dosages used in open‐label, phase 2 study (120 mg monthly with loading doses on day 8 and 15 of month 1).^7^ NTx, 25 OH‐D, bone‐specific alkaline phosphatase, comprehensive metabolic profile, and phosphorus were monitored intermittently throughout her therapy.

No adverse reactions were reported by the patient and evaluation after 1 year of treatment showed radiologic and pathologic resolution of Giant Cell Tumor of Bone (GCTB) (Fig. 1 *C*, *D*). Current dosage intervals have increased to denosumab 60 mg every 6 months.

### Case 2

**Figure 2:**
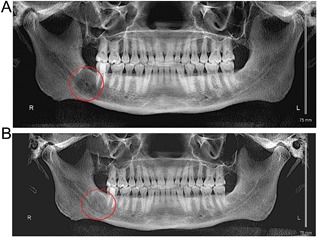
(*A*) X‐ray showing a 25 mm × 15 mm radiolucent lesion in the right posterior mandible. (*B*) Surveillance image after 1 year of treatment, which shows a denser lesion without regression in size.

A 34‐year‐old man in generally good health developed right lower jaw pain in February 2014. He had no history of calcium or other metabolic bone disorders, including Paget's disease, and was not on any chronic medications. He denied any history of radiation exposure. The patient was referred to our center by his oral surgeon who diagnosed a giant cell lesion of the jaw. Panoramic dental X‐rays showed a 25 mm × 15 mm radiolucent lesion in the right posterior mandible (Fig. 2 *A*) with biopsy confirming giant cell granuloma associated with reactive bone.

On initial workup, his calcium level was 9.5 mg/dL, PTH 12.5 pg/mL, bone‐specific alkaline phosphatase 9.4 μg/L, C‐terminal telopeptide (CTx) 70 pg/mL, and NTx 20 nM/BCE/mM creatinine, which were all within reference ranges. He had vitamin D insufficiency with a value of 22.6 ng/mL.

#### Treatment

We began supplementation with vitamin D 1000 IU daily and initiated denosumab 120 mg monthly. The treatment decision was made to start at 120 mg based on the patient's age and extent of disease. Loading doses were not administered. At 7 months of treatment, repeat imaging (Fig. 2 *B*) showed a denser lesion although there was no regression in size. Repeat biopsy 1 year after the patient's initial treatment dose showed thickened cortical bone with subjacent trabeculae exhibiting bone on bone pattern in a background of adipose tissue. There was no evidence of CGCG. NTx levels were monitored throughout the course of therapy and, given the low NTx levels, the decision was made to decrease treatment dose and increase dosing interval to denosumab 60 mg every 3 months.

### Case 3

The third patient is a 14 year‐old male with no significant personal or family history of metabolic bone disease who was found to have a mandibular jaw lesion on routine orthodontic exam. The patient denied any significant pain but did mention that he felt some “loosening of the teeth.” He had normal growth and development during childhood as reported by his mother. Pubertal development was appropriate for sex and age as well.

**Figure 3:**
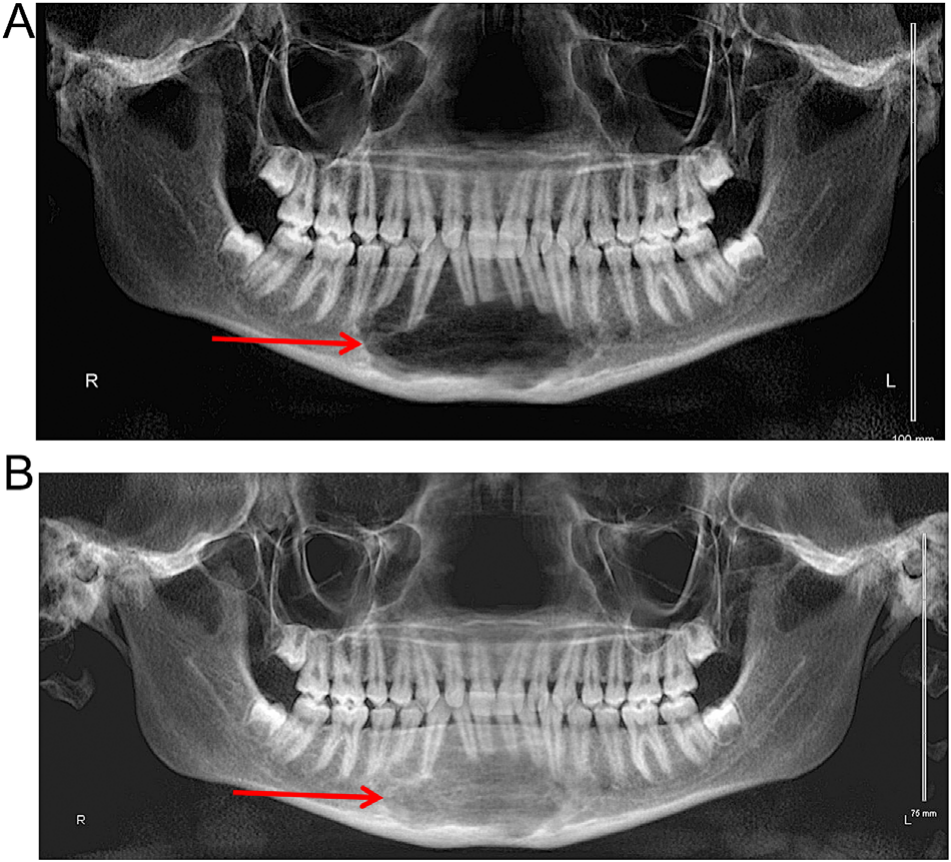
(*A*) Large expansile lesion with ill‐defined borders and a moth eaten permeative pattern. The lesion protrudes into dental roots. (*B*) One‐year image illustrates improvement in bone quality with mild reduction in size.

Panoramic dental X‐rays showed a large, destructive, expansile lesion with ill‐defined borders. There was a permeative moth‐eaten pattern indicating a more aggressive lytic lesion. The lesion measured 36 mm × 23 mm × 28 mm and protruded into the right floor of the mouth including the dental roots (Fig. 3 *A*). Based on the imaging, differential diagnosis included aggressive bone tumors such as an osteosarcoma, aneurysmal bone cyst, or a GCT. Pathology results reviewed at our institution showed bland‐appearing spindle cell proliferation with giant cells. The case was submitted to an outside expert for consultation and the final diagnosis was a giant cell granuloma.

Initial laboratory workup was within normal reference ranges except the NTx level, which was 157 nM/BCE/mM creatinine, and a 25 OH D of 14.2 ng/mL.

#### Treatment

We started treatment with denosumab 120 mg monthly. After two doses, NTx levels decreased to 14 nM/BCE/mM creatinine. The following year, repeat imaging showed marked improvement and improved bone quality (Fig. 3 *B*). He had received six consecutive doses of denosumab 120 mg every 4 weeks and interval frequency was increased to a lower dose of denosumab 60 mg every 3 months.

### Case 4

**Figure 4:**
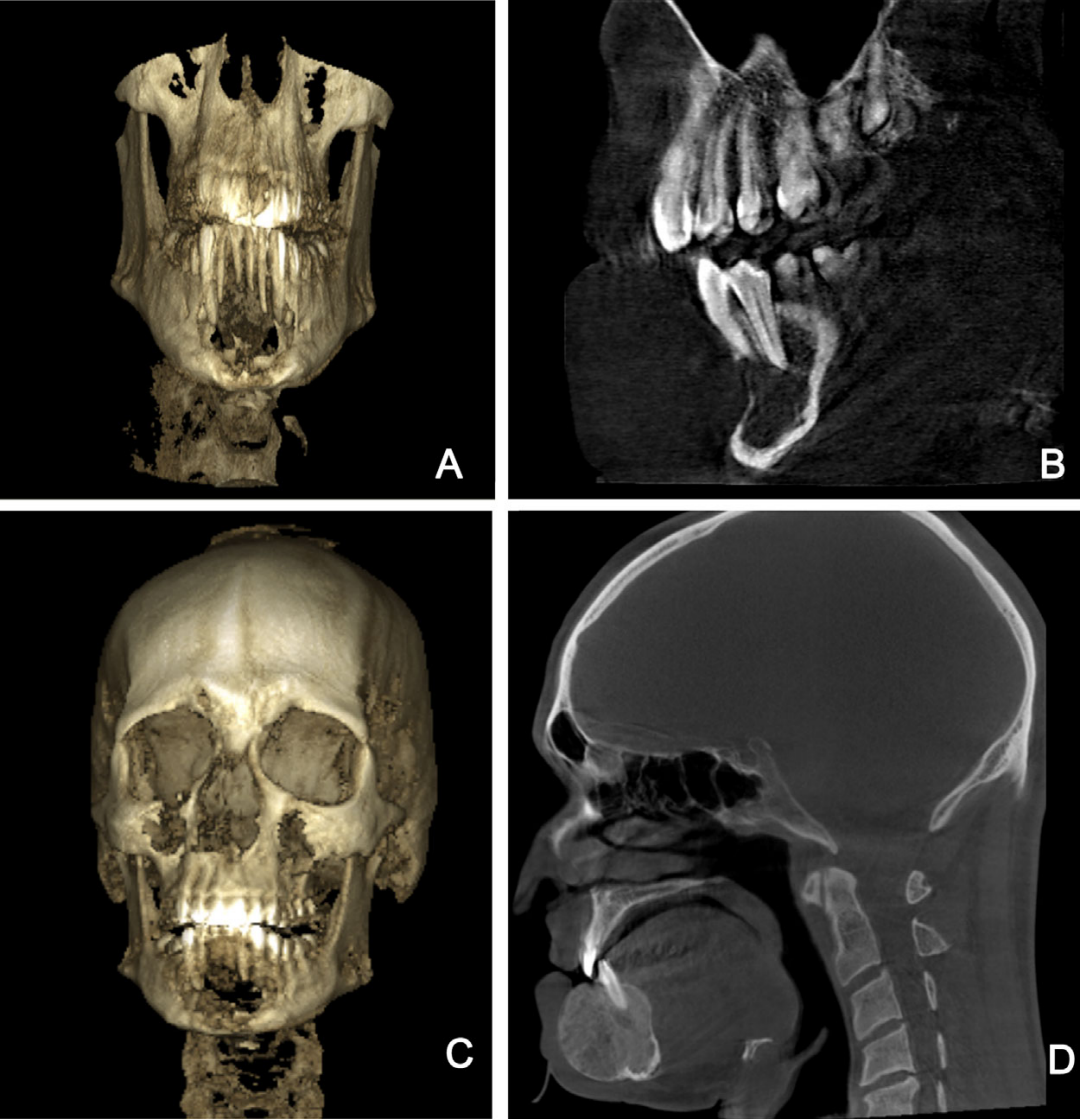
(*A*, *B*) Lytic lesion measuring 25 mm × 25 mm × 22 mm. (*C*, *D*) Surveillance imaging with 7 months of treatment showed calcification and stabilization of the lesion.

A 31‐year‐old man with no personal or family history of metabolic bone disorders was referred to our institution for evaluation and treatment of a GCT of the jaw. He was diagnosed in 2015 when he presented to his orthodontist with a lower jaw lesion and difficulty chewing food. CT scan showed an expansile lesion measuring 25 mm × 25 mm × 22 mm (Fig. 4 *A*, *B*). The lesion was described as being lytic in nature and expansile. Buccal cortex was absent and lingual cortex was almost completely absent. Prior to establishing endocrine care, he had received steroid injections with Kenalog 40 mg/mL weekly. After 6 weeks of treatment, little effect on tumor size was observed.

#### Treatment

This patient's initial laboratory workup was within normal limits and denosumab 120 mg monthly was started. He completed 7 months of treatment with no reported adverse events and surveillance imaging repeated this year showed calcifications of the lesion (Fig. 4 *C*, *D*).

### Case 5

A 15‐year‐old female with a history of presumed autosomal dominant cherubism (two sisters with a diagnosis of cherubism) and mandibular lesions since 6 years of age presented with loosening of her teeth and numbness to the lesion site. She had normal growth and development with menarche at 11 years of age. At the time of examination, she had achieved height of 5 foot 9 inches without growth hormone treatment—reportedly taller than her parents or siblings. The patient was diagnosed as having GCT of the jaw with concomitant enlargement of the mandible. Pathology confirmed giant cell lesion consistent with cherubism.

Cherubism is a rare benign condition characterized by bilateral expansion of the mandible and/or maxilla that becomes noticeable within the first several years of life. It becomes progressively pronounced until puberty, with gradual involution by middle age. Histologically, the lesions contain numerous multinucleated giant cells scattered throughout a fibrous tissue stroma. In some cases, lesions resolve without treatment. However, the frequency of occurrence is unknown because most cases have been surgically treated before reaching puberty. For patients with extensive lesions and risk of fracture, segmental mandibulectomy followed by reconstruction with a fibular flap has been suggested. Medical therapy including calcitonin and low‐dose interferon alpha have also been used.^8^

#### Treatment

The decision was made to start denosumab 120 mg monthly because the patient had progressive lytic lesions and numbness with core biopsy revealing giant cell pathology. Further, we considered investigating genetic testing for mutations in the SH3 binding protein (SH3PB2) on chromosome 4p16.3,^9^ which causes cherubism, but did not pursue due to accessibility of the test, as well as it having little direct clinical impact on our management.

After 5 months of treatment, dental imaging showed significant ossification of the giant cell defects. A second biopsy to confirm response to therapy is pending.

### Case 6

A 19‐year‐old female with a previous dental implant who presented to her orthodontist with related complications, and biopsy confirmed CGCG of the jaw.

#### Treatment

Patient is tolerating denosumab 120 mg monthly. Surveillance is ongoing, imaging and repeat biopsy are planned for the 1‐year treatment mark.

### Case 7

A 12‐year‐old male with CGCG of the jaw post presented to our care after resection of a 2‐cm lesion with recurrence.

#### Treatment

Treatment was initiated with denosumab 60 mg, which was determined based on shared‐decision making with family, who had concerns that higher doses may affect growth and bone quality during formative pubertal/developmental years. The patient received a single dose of medication then developed paresthesia and back pain 1 month later. Pretreatment calcium was 9.9 mg/dL, PTH 63 pg/mL, and 25 OH D 20.2 ng/mL while he was on supplementation with vitamin D 1000 units daily. On laboratory evaluation for his acute symptoms, the patient was found to have secondary hyperparathyroidism with a serum calcium of 6.3 mg/dL (albumin of 4.6 g/dL), PTH of 292 pg/mL, and 25 OH D 23.4 ng/mL. Calcium 600 mg three times a day was started along with ergocalciferol 50,000 units weekly. Denosumab was held and paresthesia and back pain have improved. The most recent calcium level was 9.6 mg/dL (albumin of 4.6 g/dL) and family is considering restarting treatment with denosumab at lower doses.

Table 1 is a summary of the characteristics of the seven cases discussed herein.

**Table 1:** Patient Characteristics

Patient	Age (years)	Calcium (mg/dL)	PTH (pg/mL)	25‐OH vitamin D (ng/mL)	Bone‐specific alkaline phosphatase (mc/L)	NTx (nM BCE/mM creatinine)
1	20	9.9	17	9.8	13	71
2	34	9.9	NA	22.6	9.4	20
3	14	10.7	31	14.2	43	157
4	31	10.1	28	29.3	NA	31
5	15	9.8	44	7.4	NA	NA
6	19	10	28	29.8	18	NA
7	12	9.9	63	20.2	106	NA

NA = not available.

## Results and Discussion

To date, there are several single‐case reports, but lack of aggregate data, documenting success with denosumab for the treatment of CGCG and GCT affecting the jaw. The FDA‐approved doses for long bone lesions, denosumab 120 mg every 4 weeks with loading doses on days 8 and 15, may be too high and lead to more toxicity, especially in younger patients. Schreuder and colleagues^10^ describe a case report of a 25‐year‐old woman with type 1 diabetes with progressive swelling of her anterior maxilla. She was found to have a biopsy confirmed diagnosis of CGCG. Her clinical exam included severe protrusion of the upper lip and flattening of nasolabial folds. The patient was initiated on calcitonin nasal spray 200 IU daily followed by calcitonin 100 IU subcutaneously and additive treatment with pegylated interferon for progressive growth. The patient was scheduled for subtotal maxillectomy and sought a second opinion. The authors initiated treatment with denosumab 120 mg with loading doses on day 8 and day 15 of month 1. Two months after therapy, the patient noticed less mobility and CT scan at 6 months demonstrated regression of the tumor, reformation of the cortex along the periphery, and intralesional bone formation. We report here four cases of successful treatment of CGCG and GCT of the jaw, using both low and high doses of denosumab. The first two cases were followed to resolution of the disease based on confirmed biopsy, and cases 3 and 4 show promising response on imaging.

Traditionally, the treatment for CGCG is surgical resection. The extent of tissue removal ranges from simple curettage to en bloc resection. Unfortunately, surgery can be associated with recurrence and serious facial mutilation. Loss of teeth and tooth germs are an unavoidable consequence.^11^ Medical treatment alternatives would therefore be preferential if tumor regression or improvement of bone quality can be achieved.

As shown by our case series, patients are presenting at a younger age, which requires providers to use clinical acumen when recommending treatment. The effects of FDA‐approved doses of denosumab in GCT of the long bones may not be appropriate for adolescent patients who may not have reached skeletal maturity. Finally, potential adverse reactions must be monitored. There have been two cases of GCT of the long bone transforming to osteosarcoma while on treatment with denosumab at FDA‐approved doses.^12^ In the original phase‐2 study, three patients had new primary malignancies—two had sarcoma (of which one was retrospectively suspected to be present at baseline and the other was attributed to be malignant transformation) and one had thyroid cancer.^13^ We observed hypocalcemia in one case that was alleviated by oral supplementation. There were no reported cases of infection.

This analysis gives us a qualitative sense that denosumab, at both high and low doses, is an effective treatment option for CGCG and GCT of the jaw. Surgery was forestalled in our case series, meeting our primary clinical aim. Based on the surveillance imaging that we have to date, our secondary endpoint of radiologic improvement was also reached. We recognize the need for further studies that will elucidate quantitative measures in monitoring clinical outcomes. As of now, the off‐effect of denosumab therapy on these lesions is unknown. We recommend that doses and dosage frequency/intervals be considered on an individual patient basis. Bone turnover markers, calcium and vitamin D levels, dental imaging/biopsies, and bone densitometry measured at baseline and appropriate intervals provide clinical support when determining duration of treatment.

## Disclosures

The authors state they have no conflicts of interest, except Salvatore Ruggiero, who is a consultant for Amgen.
